# Comparison of the main components and bioactivity of *Rhus verniciflua Stokes extracts* by different detoxification processing methods

**DOI:** 10.1186/s12906-018-2310-x

**Published:** 2018-08-30

**Authors:** Seon-Ok Lee, Sung-Ji Kim, Ju-Sung Kim, Hyuk Ji, Eun-Ok Lee, Hyo-Jeong Lee

**Affiliations:** 1 Department of Science in Korean Medicine, Graduate School, Kyung Hee University, Hoegi-dong, Dongdaemun-gu, 130-701 Seoul, Republic of Korea; 2 Department of Cancer Preventive Material Development, Graduate School, Kyung Hee University, Hoegi-dong, Dongdaemun-gu, 130-701 Seoul, Republic of Korea; 3 College of Korean Medicine, Kyung Hee university, 1 Hoegi-dong, Dondaemun-gu, 130-701 Seoul, Republic of Korea; 4 Major of Plant Resources and Environment, College of Applied Life Sciences, 102 Jeju National University, Jeju-si, Jeju-do 690-756 Korea

**Keywords:** *Rhus verniciflua Stokes*, Detoxification, Fermentation, Fustin, Fisetin

## Abstract

**Background:**

*Rhus verniciflua* Stokes is an Asian tree species that is used as a food supplement and traditional medicine in Korea. However, its use is restricted by its potential to cause allergy. Thus, allergen-free *R. verniciflua* extracts are currently being marketed as a functional health food in Korea. In the present study, three different allergen-free *R. verniciflua* extracts (DRVE, FRVE, and FFRVE) were produced by detoxification of *R. verniciflua,* and their properties and constituents were compared.

**Methods:**

The main components and properties (antibacterial, antioxidant, anticancer, and hepatic lipogenesis inhibitory effects) of the three allergen-free extracts were compared. Moreover, the major phenolic constituents of *R. verniciflua*, including gallic acid, fustin, fisetin, and quercetin, were analyzed in the three extracts.

**Results:**

DRVE was superior to the two other extracts with regard to antioxidant activity, while FRVE was superior with regard to antimicrobial activity and suppression of hepatic lipogenesis. FRVE exhibited lipid-lowering effects by lowering sterol regulatory element-binding protein 1 and triglyceride levels, and promoting the activation of peroxisome proliferator-activated receptor and AMP-activated protein kinase in an in vitro model of non-alcoholic fatty liver.

**Conclusions:**

Overall, our findings demonstrate various differences among the three extracts. This suggests that functional and bioactive compounds present in *R. verniciflua* could be altered by the detoxification process, and this property could be considered in the development of functional health foods in the future.

## Background

*Rhus verniciflua* Stokes (RVS), the lacquer tree, has been used as a traditional medicine and food supplement for a long time in Eastern Asia. In Korea, RVS has been used as a herbal medicine for the treatment of abdominal pain, including pain caused by stomach disorders such as gastritis, and as a hemostatic agent [1]. RVS has been reported to exhibit anticancer, antioxidant, antimicrobial, and anti-inflammatory activities [2–6]. Moreover, RVS contains a wide variety of flavonoids and polyphenols, including fustin, fisetin, quercetin, butein, p-coumaric acid, kaempferol, sulfuretin, catechol, and ethyl gallate. However, despite the various biological activities of RVS, its use has been limited because of a component called urushiol, which is known to cause allergies. Therefore, urushiol should be removed before using RVS as a food supplement or medicine. Several detoxification methods have been developed to produce urushiol-free RVS, such as heat treatment, solvent extraction, and enzyme treatment by microbial or mushroom mycelium fermentation [7–9]. Currently, allergen-free RVS extracts are being marketed as health functional food in Korea. However, there are no comparative studies on the components and bioactivities of RVS extracts obtained by various detoxification methods. It is important to develop efficient and cost-effective food processing methods in order to enhance the content of bioactive components.

We previously reported that RVS detoxified via a microbial method could alleviate oleic acid (OA)-induced steatosis in HepG2 cells, and that it contained phenolics and cosanols with lipid-lowering potential. In the present study, we compared the effects of three RVS extracts detoxified by different methods with regard to their antioxidant, antimicrobial, and anticancer properties, their suppressive effect on hepatic lipogenesis, and their main components.

## Methods

### Preparation of DRVE, FRVE, and FFRVE powders

The three types of detoxified RVS extracts used in this study are products that are commercially available in Korea, and were purchased from Okkane (Seoul, Korea). The following three extracts were used: an allergen-free RVS extract detoxified by hot air drying (dried *R. verniciflua* extract, DRVE), an allergen-free RVS extract fermented with *Saccharomyces carlsbergensis* (fermented *R. verniciflua* extract, FRVE), and an allergen-free RVS extract fermented with mushroom mycelium (*Fomitella fraxinea*-fermented *R. verniciflua* extract, FFRVE). The extracts (one bottle = 1.5 L) were dried to a powder by freeze-drying.

### Determination of antimicrobial activity by agar diffusion method

The antimicrobial activities of the three RVS extracts were determined via modified Kirby-Bauer disk diffusion method [10, 11]. The test microorganisms used in this experiment were *Propionibacterium acnes* (ATCC 6919) and *Trichophyton rubrum* (ATCC 22402), and were obtained from the Korean Culture Center of Microorganisms. *P. acnes* was cultured anaerobically at 37 °C in Mueller Hinton broth (Difco, USA) and Mueller Hinton agar (0.75% agar), while *T. rubrum* was cultured at 26 °C in Sabouraud Dextrose broth and Sabouraud Dextrose agar (0.75% agar). The three powdered extracts, DRVE, FRVE, and FFRVE, were dissolved in water to obtain concentrations of 100 and 1000 mg/mL for each extract. Fifty microliters of each extract was injected into a sterile disk of 6-mm diameter (Toyo Roshi Kaisha Ltd., Tokyo, Japan), and the solvent was allowed to dry off in an aseptic hood. Accordingly, disks were loaded with 5 and 50 mg of each crude extract. Standard disks containing distilled water served as negative controls for the antimicrobial test.

After 24 h, *P. acnes* or *T. rubrum* cultures were adjusted to 1 × 10^8^ CFU/mL using 0.5 McFarland standards and inoculated into Mueller Hinton agar and Sabouraud Dextrose agar, respectively. Next, disks containing the extracts were placed on a plate and incubated at 37 and 26 °C, respectively. *P. acnes* and *T. rubrum* plates were incubated for 2 and 5 days, respectively. The antimicrobial activities of the extracts were then determined by measuring the diameter of the clear zones around the disks in millimeters. This measurement was carried out in triplicate.

### Cell culture

Human hepatocellular carcinoma (HepG2, ATCC HB-8065) and murine normal hepatocyte (AML12, ATCC CRL-2254) cell lines were maintained in Dulbecco’s modified Eagle’s medium (DMEM) containing 10% fetal bovine serum (FBS, Welgene, Daegu, Korea) with 1% antibiotics (Welgene). Human prostate cancer (PC3, ATCC CRL-1435), breast cancer (MDA-MB-231, ATCC HTB-26), and colon cancer (HCT116, ATCC CCL-247) cell lines were maintained in RPMI 1640 containing 10% FBS (Welgene) with 1% antibiotics (Welgene).

### Cell viability assay

HepG2, PC3, MDA-MB-231, HCT116, and AML12 cells were incubated in 96-well plates (5 × 10^4^ cells/well) and treated with various doses of DRVE, FRVE, and FFRVE (0, 50, 100, 200, and 400 μg/mL) for 24 h. Next, 50 μL of 3-(4–5-dimethylthiazol-2-yl)-2,5-diphenyl tetrazolium bromide (MTT, 1 mg/mL, Sigma-Aldrich, St. Louis, MO, USA) was added. After 2 h, formazan crystals in viable cells were dissolved in dimethyl sulfoxide (DMSO), and cell viability was calculated by measuring the optical density (OD) at 570 nm using a microplate reader (Molecular Devices Co, Sunnyvale, CA, USA).

### High-performance liquid chromatography

Urushiol in the three extracts was analyzed by high-performance liquid chromatography (HPLC, Agilent Technologies, Santa Clara, CA, USA) using Hichrome HPLC columns (5 μm, 250 mm × 4.6 mm, Hichrome, Ltd., Theale, UK). A flow rate of 0.3 mL/min and an injection volume of 10 μL were used. The solvent used was 100% methanol, and the detection wavelength was set to 254 nm.

Moreover, the polyphenols gallic acid, fustin, fisetin, quercetin, butein, and sulfuretin were analyzed in the three RVS extracts via HPLC. The mobile phases were composed of 0.1% formic acid in water (solvent A) and 100% methanol (solvent B), delivered at a flow rate of 0.7 mL/min. The following gradient conditions were used: 0–17 min, 100% B; 17–20 min, 100% B; 20–23 min, 0% B; and 23–30 min, 0% B. The detection wavelength was set to 254 nm, and an injection volume of 10 μL was used.

### Oil red O staining

HepG2 cells were incubated in a 6-well plate (5 × 10^5^ cells/well) for 24 h. The cells were then treated with DRVE, FRVE, and FFRVE (400 μg/mL), and stimulated with OA (200 μM) for a further 24 h. After incubation, the supernatants were discarded and cells were washed with phosphate-buffered saline (PBS). Cell fixation was done by treating with 4% formalin for 10 min, followed by washing with PBS and staining with Oil Red O (ORO). ORO stain was extracted using isopropanol and the OD was measured at 510 nm using a microplate reader (Molecular Devices Co).

### Western blot analysis

HepG2 cells were incubated in a 6-well plate (5 × 10^5^ cells/well) for 24 h. The cells were then treated with DRVE, FRVE, and FFRVE (400 μg/mL), and stimulated with OA (200 μM) for a further 24 h at 37 °C. Next, cells were harvested and lysed with radioimmunoprecipitation assay (RIPA) buffer (50 mM Tris-HCl (pH 7.4), 150 mM NaCl, 1% NP-40, 0.25% sodium deoxycholate, 1 M EDTA, 1 mM Na_3_VO_4_, 1 mM NaF, and protease-inhibitor cocktail). Protein samples were quantitated using a Bio-Rad DC protein assay kit II (Bio-Rad, Hercules, CA, USA), resolved via sodium dodecyl sulfate-polyacrylamide gel electrophoresis (SDS-PAGE) on 8% polyacrylamide gel, and electrotransferred onto a BioTrace NT transfer membrane (Pall, Gelman Laboratory, Port Washington, NY, USA). After electrotransfer, membranes were blocked with 5% skimmed milk (BD, NJ, USA) and probed with primary antibodies for sterol regulatory element-binding protein 1 (SREBP-1, Santa Cruz Biotechnology, Santa Cruz, CA, USA), peroxisome proliferator-activated receptor alpha (PPAR-α, Santa Cruz Biotechnology), AMP-activated protein kinase (AMPK, Cell Signaling, Denvers, MA, USA), phosphorylated AMPK (p-AMPK, Cell Signaling), and β-actin (Sigma-Aldrich) overnight, and exposed to horseradish peroxidase-conjugated secondary anti-mouse or anti-rabbit antibodies. Protein expression levels were detected using an EZ-Western Lumi Pico kit (DOGEN, Seoul, Korea).

### Measurement of cellular triglyceride levels

HepG2 cells were incubated in a 6-well plate (5 × 10^5^ cells/well) for 24 h. The cells were then treated with DRVE, FRVE, and FFRVE (400 μg/mL), and stimulated with OA (200 μM) for a further 24 h. To measure cellular triglyceride (TG) levels, a chloroform-methanol extraction method was applied with some modifications, as described in a previous protocol [12, 13]. Cells were collected and mixed with 1 mL of a 2:1 chloroform:methanol mixture at room temperature for 20 min. After centrifugation at 500×*g* for 10 min, the lower layers were collected and dried overnight at 4 °C, and TG levels were measured using a TG assay kit (Asan Pharm, Hwaseong-si, Korea). The OD was measured at 550 nm using a microplate reader (Molecular Devices Co).

### Determination of the antioxidant capacity of the three extracts

The 1,1-diphenyl-2-picrylhydrazyl (DPPH) radical scavenging activity was measured by the Blois method [14]. The extracts were mixed with DPPH and stabilized at room temperature for 30 min. The OD was then measured at 515 nm using a microplate reader (Molecular Devices Co).

### Statistical analysis

Results are presented as means ± standard deviation of triplicates. Data were analyzed via Student’s *t*-test. Differences among groups were considered statistically significant if *p* < 0.05.

## Results

### Confirmation of DRVE, FRVE, and FFRVE detoxification

**Fig. 1:**
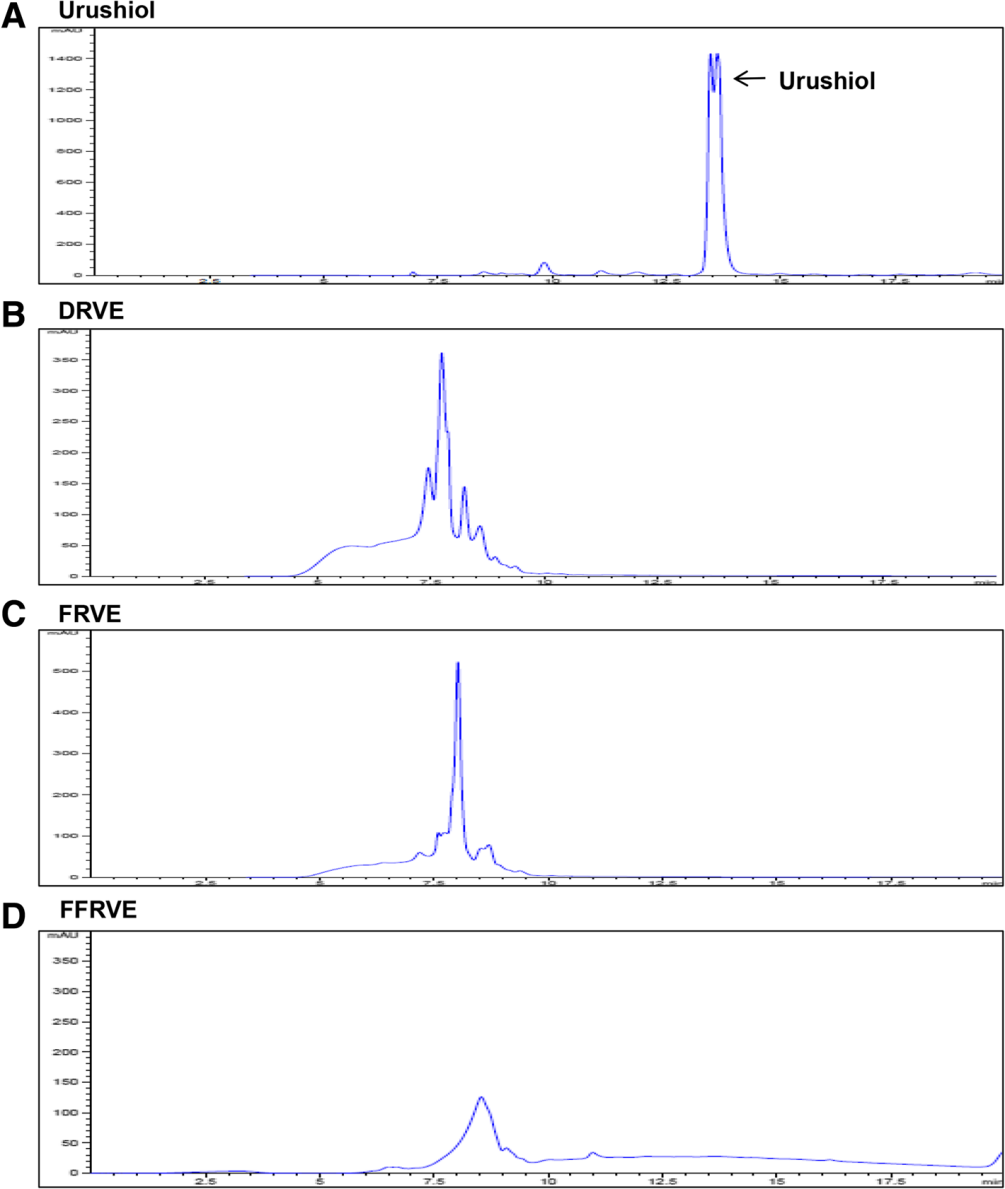
Determination of allergen-free *Rhus verniciflua Stokes* extracts (DRVE, FRVE, FFRVE) by HPLC analysis. HPLC analysis identified urushiol as an allergen of *Rhus verinciflua Stokes*. **a** Urushiol standard **b** DRVE **c** FRVE **d** FFRVE

To confirm detoxification of the three RVS extracts, the presence of the allergen urushiol was investigated by HPLC. Urushiol (Fig. 1a) was not detected in any of the three extracts (Fig. 1b, c, and d), thus confirming successful detoxification. The urushiol peak in the standard appeared at 13.62 min.

### DRVE demonstrated the strongest antioxidant activity among the three extracts

The antioxidant activities of DRVE, FRVE, and FFRVE were evaluated via a DPPH radical scavenging assay. Quercetin, tannic acid, and ascorbic acid were used as standards to analyze DPPH scavenging capacity.

The half-maximal effective concentration (EC_50_) of any antioxidant compound is inversely related to its antioxidant activity, as it is the concentration of the antioxidant needed to decrease the radical concentration by 50%. Thus, lower EC_50_ values indicate higher antioxidant activities. DRVE and FRVE exhibited EC_50_ values of 48.7 and 111.3 μg/mL, respectively, while the DPPH radical scavenging activity of FFRVE was 28.6% at its high concentration (200 μg/mL, Table 1). The radical scavenging activity of DRVE was stronger than that of the two other extracts (Table 1).

**Table 1:** Antioxidant capacity of the DRVE, FRVE, and FFRVE by DPPH scavenging assay

Sample	DPPH, EC50 (μg/ml)
DRVE	48.7 ± 2.1
FRVE	111.3 ± 1.3
FFRVE	> 200 (28.6%)
Quercetin	7.7 ± 0.1
Tannic acid	8.4 ± 0.5
Ascorbic acid	11.8 ± 1.1

Each value represents mean ± SD (*n* = 3)

### FRVE demonstrated the strongest antimicrobial activity among the three extracts

**Fig. 2:**
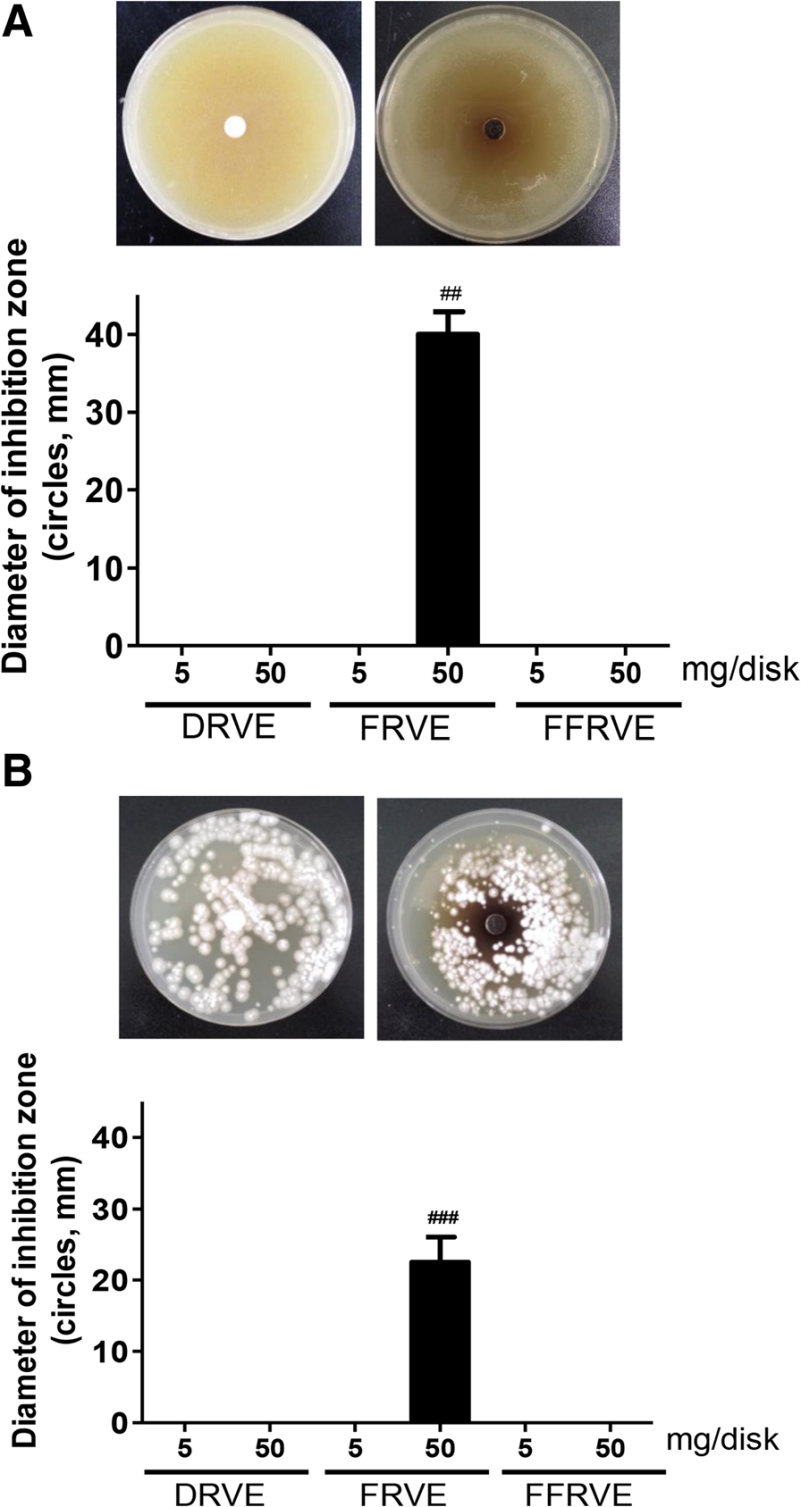
Antimicrobial activity of DRVE, FRVE and FFRVE. Inhibitory growth zone of DRVE, FRVE and FFRVE against (**a**) *Propionibaterium acnes* and (**b**) *Trichphyton rubrum.* Err bars indicate standard deviations. All experiments were duplicates*.* *** represent significant differences (*p* < 0.001) with respect to the control (pathogen only)

The antimicrobial activities of the three RVS extracts were investigated against two different microbes that cause skin diseases (*P. acne*, an acne-causing bacterium, and *T. rubrum*, a fungus that affects hands and nails and causes athlete’s foot) using an agar diffusion method at doses of 5 and 50 mg/disk. Only the 50 mg/disk dose of FRVE demonstrated a strong antimicrobial activity against *P. acnes* (Fig. 2a) and *T. rubrum* cultures (Fig. 2b), producing 42- and 25-mm inhibition zones, respectively*.*

### The anticancer effects of DRVE, FRVE, and FFRVE

#### DRVE and FRVE inhibit cancer cell viability without affecting normal cells

We tested the effects of DRVE, FRVE, and FFRVE on the viability of four human cancer cell lines (HepG2, PC3, MDA-MB-231, and HCT116) and of normal cells (AML-12). The concentrations of FRVE and DRVE tested were 0, 50, 100, 200, and 400 μg/mL.

**Fig. 3:**
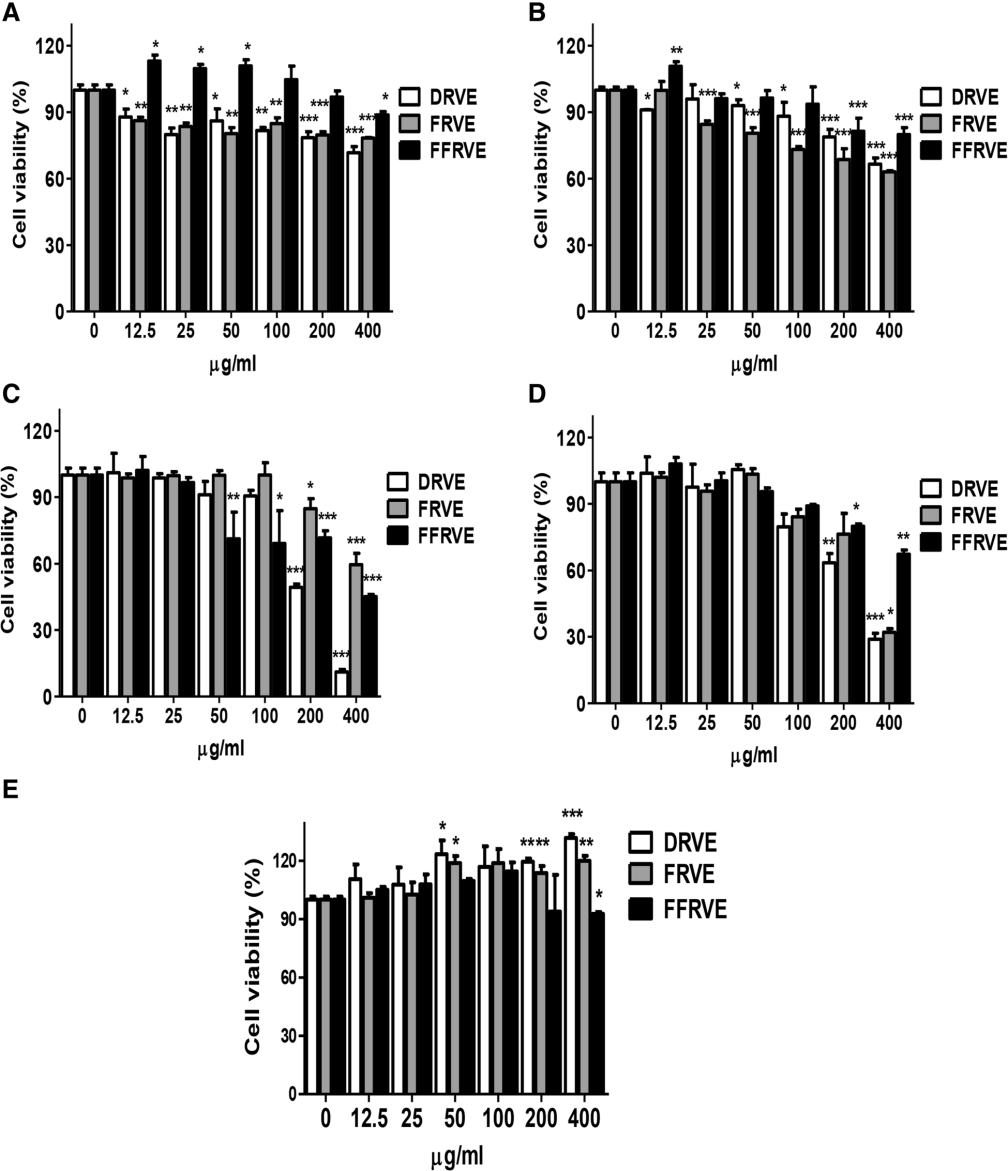
Cell viability of DRVE, FRVE and FFRVE against various cancer cells. Cells were treated with various concentrations of DRVE, FRVE and FFRVE (0, 12.5, 25, 50, 100, 200, and 400 μg/ml) for 24 h. **a** HepG2, **b** PC-3, **c** MDA-MB-231, **d** HCT-116, **e** AML-12 cells. Data are represented as the mean ± SD for three experiments. * *p* < 0.05, ** *p* < 0.01, and *** *p* < 0.001 (in comparison to the control)

As shown in Fig. 3a, the viability of HepG2 cells was reduced to 29, 22, and 12% when incubated with 400 μg/mL DRVE, FRVE, and FFRVE, respectively, for 24 h (Fig. 3a). Similarly, treatment with 400 μg/mL DRVE, FRVE, and FFRVE reduced prostate cancer cell viability by 39, 37, and 21%, respectively (Fig. 3b). Of all the cancer cell lines studied, the breast cancer cell line MDA-MB-231 showed the lowest viability (11%) after treatment with 400 μg/mL DRVE (Fig. 3c). With regard to colorectal cancer cells, DRVE and FRVE treatments showed similar effects on the viabilities of HCT116 cells (28 and 31%, respectively), whereas viability was approximately twice greater after FFRVE treatment (67%, Fig. 3d). Overall, DRVE exhibited the strongest cytotoxic effect against cancer cells.

In contrast, DRVE and FRVE did not affect the viability of normal cells (AML-12), even at the highest concentration tested (Fig. 3e), while 400 μg/mL FFRVE inhibited the growth of AML-12 cells by 8% (Fig. 3e).

#### The three RVS extracts suppressed hepatic lipogenesis in an in vitro model of non-alcoholic fatty liver

**Fig. 4:**
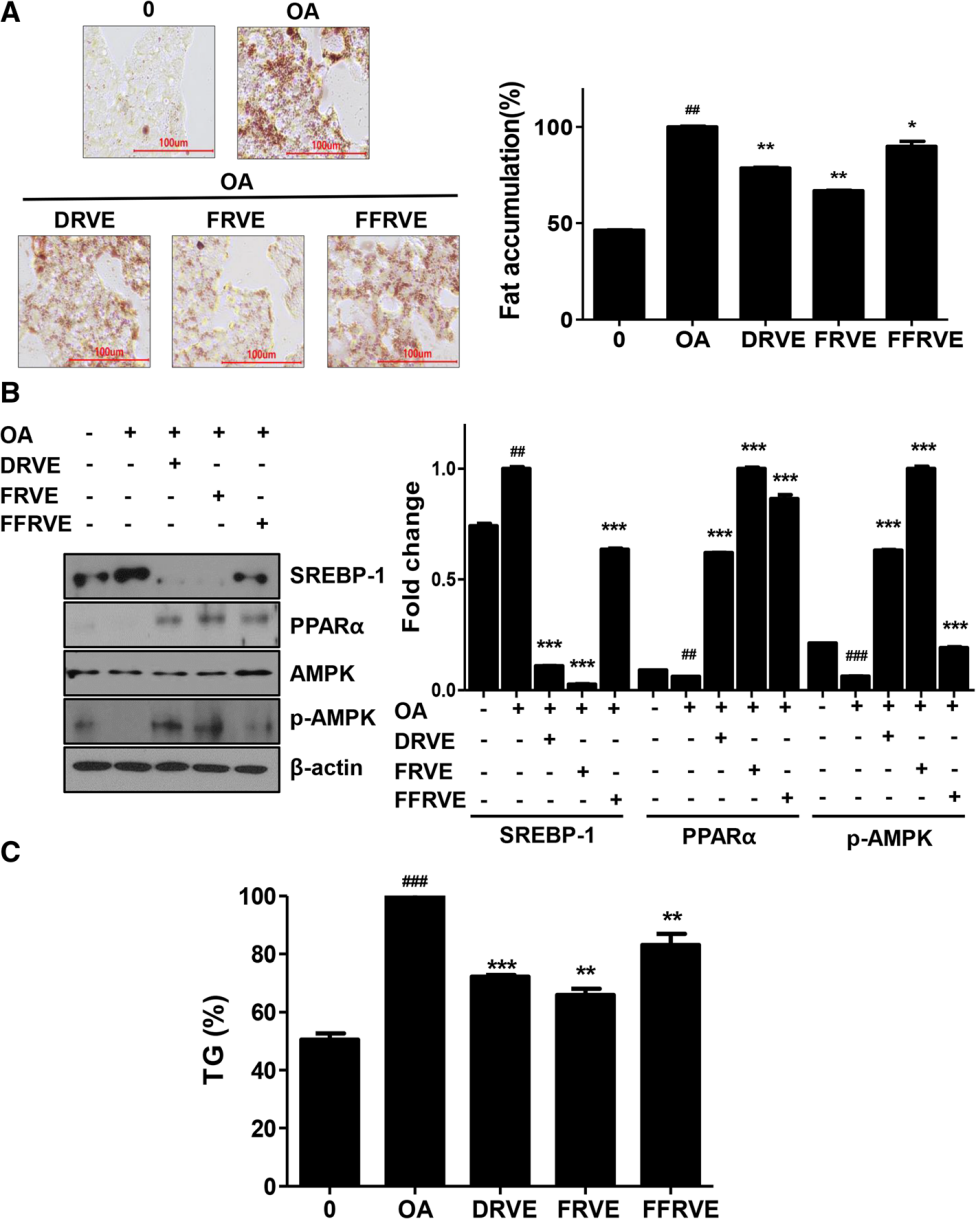
Effect of DRVE, FRVE, FFRVE on cellular lipid accumulation and lipogenesis in OA-induced HepG2 cells. Cells were co-treated with OA and 400 μg/ml of DRVE, FRVE, FFRVE for 24 h. **a** Cells were stained with ORO as described in the materials and methods and then quantitatively analyzed. ORO staining image (magnification 400×), (##) *p* < 0.01 (in comparison to the non-OA treated control), * and ** represent significant differences (*p* < 0.05 and *p* < 0.01, respectively) with respect to the OA-treated control. **b** Determination of the expression of AMPK, SREBP-1 and PPARα by western blotting analysis. Quantitative protein levels were shown. (##) *p* < 0.01, and (###) *p* < 0.001 (in comparison to the non-OA-treated control). (∗∗) *p* < 0.01 and (∗∗∗) *p* < 0.001 (in comparison to the OA-treated control). **c** Total intracellular TG was analyzed by an enzymatic colorimetric method. Data are represented as the mean ± SD of three experiments. (###) *p* < 0.001 (in comparison to the non-OA treated control). (∗∗) *p* < 0.01 and (∗∗∗) *p* < 0.001 (in comparison to the OA-treated control)

We previously reported that FRVE could inhibit hepatic lipogenesis in OA-treated HepG2 cells. In order to investigate whether DRVE and FFRVE could alleviate OA-induced cellular steatosis, OA-treated HepG2 cells were stained with ORO. As shown in Fig. 4a, DRVE decreased the number and size of lipid droplets in OA-treated HepG2 cells. DRVE, FRVE, and FFRVE treatments decreased the fat deposits by 22, 34, and 11%, respectively, compared with that in OA-treated HepG2 cells not treated with extracts (Fig. 4a).

To evaluate the regulatory effect of the extracts on lipogenesis and fatty acid oxidation, the levels of SREBP-1, PPAR-α, and AMPK in HepG2 cells were determined via western blotting. Our results showed that the three RVS extracts downregulated OA-induced elevation of SREBP-1 levels in HepG2 cells. FRVE and DRVE increased the expression of PPAR-α and p-AMPK, while FFRVE increased PPAR-α but not p-AMPK expression levels (Fig. 4b). In order to assess OA-induced TG accumulation, and the effects of the extracts on it, cellular TG levels were measured. OA-treated cells exhibited elevated TG levels compared to non-treated cells. However, treatment with DRVE, FRVE, and FFRVE lowered TG levels to 28, 35, and 17%, respectively (Fig. 4c).

Although the three extracts demonstrated lipid-lowering effects in the in vitro model of non-alcoholic fatty liver, DRVE and FRVE were more effective than FFRVE at the same concentration.

#### Comparison of the polyphenol content in the three RVS extracts

**Fig. 5:**
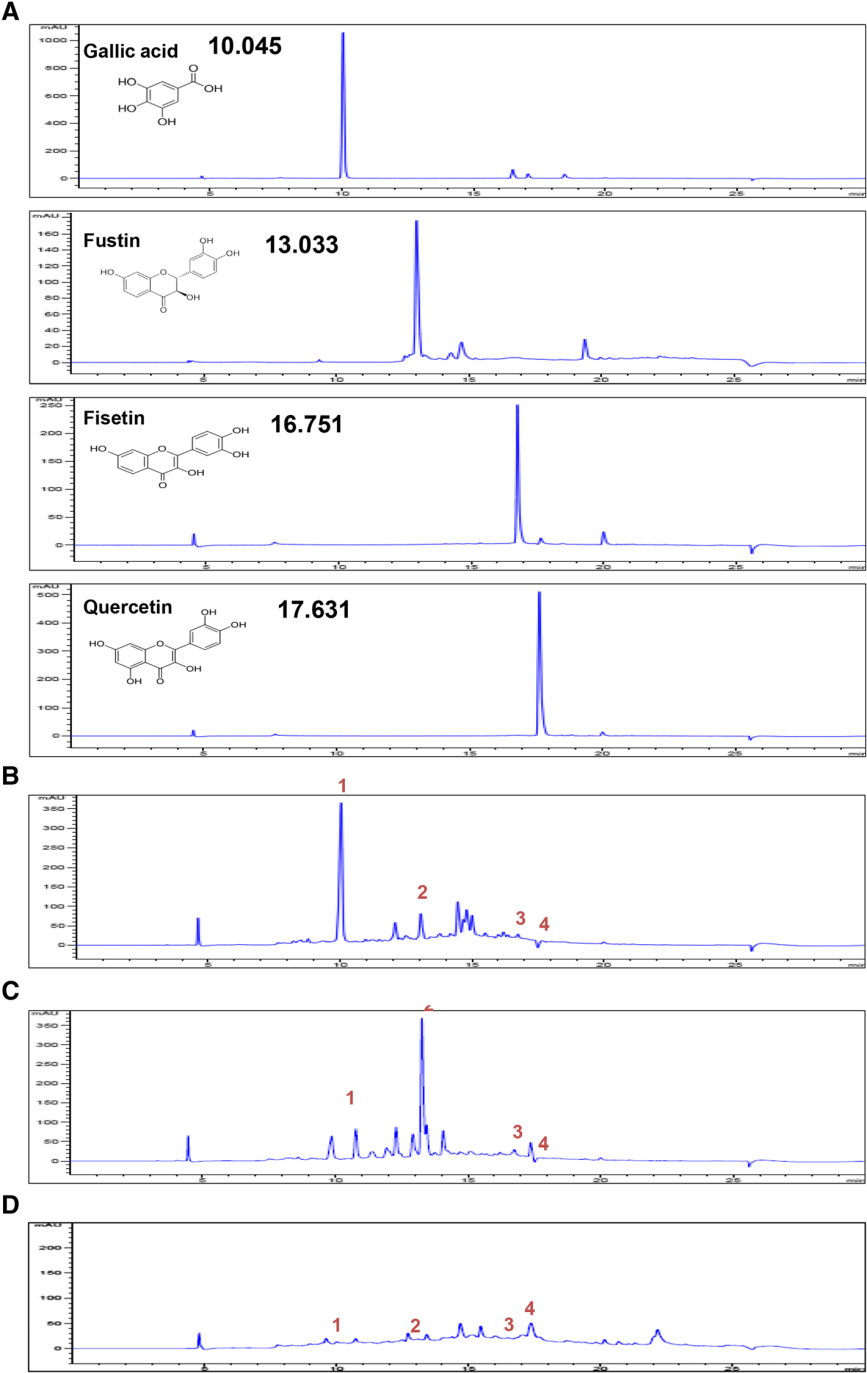
HPLC chromatograms of the DRVE, FRVE, and FFRVE. Peaks of four main components (**a**) are gallic acid (1), fustin (2), fisetin (3), quercetin (4) and DRVE (**b**), FRVE (**c**), FFRVE (**d**). Mobile phase: 0.1% (*v*/v) Formic acid in water (solvent A) and 100% (v/v) Methanol (solvent B) at a flow rate of 0.7 ml/min, with gradient as follows: 0–17 min, 100%B; 17–20 min, 100%B; 20–23 min, 0%B; 23–30 min, 0%B. and detected at 254 nm

Gallic acid, fustin, fisetin, quercetin, butein, and sulfuretin are the main active constituents of *R. verniciflua.* We compared the contents of four phenolic compounds (gallic acid, fustin, fisetin, and quercetin) as marker compounds in DRVE, FRVE, and FFRVE via HPLC. The retention times of gallic acid, fisetin, fustin, and quercetin were 10.045, 13.033, 16.751, and 17.631 min, respectively (Fig. 5a).

These polyphenols were detected in DRVE, FRVE, and FFRVE eluents (Fig. 5b, c, and d, respectively). Gallic acid levels in DRVE, FRVE, and FFRVE were 170, 33, and 4 mg/g, respectively, and gallic acid content in DRVE was therefore 42.5 times greater than that in FFRVE (Table 2). Fustin levels in DRVE, FRVE, and FFRVE were 34.5, 129, and 10.7 mg/g, respectively, and fustin content in FRVE was therefore 12 times higher than that in FFRVE (Table 2). Fisetin levels in DRVE, FRVE, and FFRVE were 15, 59, and 1 mg/g, respectively, and fisetin content was therefore 59 times greater in FRVE than in FFRVE. Finally, quercetin levels in DRVE, FRVE, and FFRVE were 1.5, 4, and 19 mg/g, respectively (Table 2).

**Table 2:** Contents of Phenolic acids in DRVE, FRVE, and FFRVE

Sample	Compound (mg/g)			
DRVE	Gallic acid	Fustin	Fisetin	Quercetin
FRVE	170	34.5	15	1.5
FFRVE	33	129	59	4
Sample	4.3	10.7	19	7

#### The four marker compounds of DRVE, FRVE, and FFRVE possess antioxidant activities

According to the DPPH scavenging assay, the antioxidant activities of the evaluated compounds could be ordered as follows: fisetin > gallic acid > ascorbic acid > quercetin > fustin (Table 3).

**Table 3:** Antioxidant effect of four main compounds by DPPH scavenging assay

Sample	DPPH, EC50 (μg/ml)
Ascorbic acid	8.1 ± 0.6
Gallic acid	1.8 ± 0.1
Fustin	15.1 ± 0.0
Fisetin	1 ± 0.0
Quercetin	7.9 ± 0.2

Each value represents mean ± SD (*n* = 3)

Fisetin showed the strongest radical scavenging activity and the lowest EC_50_ value (1 ± 0.01 μg/mL), whereas fustin exhibited the lowest DPPH scavenging activity and the highest EC_50_ value (15.1 ± 0.04 μg/mL, Table 3).

The relative antioxidant capacities of DRVE and FRVE (Table 1) were similar to those of gallic acid and fustin, respectively.

## Discussion

Despite the various benefits of RVS, it is known to cause allergy, which may be due to the presence of urushiol. Nevertheless, due to its numerous biological activities, RVS is of high importance in the development of functional food and medicine. Therefore, various detoxification methods have been developed for the removal of allergens from RVS. In Korea, the Ministry of Food and Drug Safety permitted the use of detoxified RVS extracts in 2012. Since then, the types of functional foods containing detoxified RVS extracts have been steadily increasing on a yearly basis. Detoxification methods include the removal of allergens from RVS using solvents [15], electron beam radiation [16], high temperature [17], and microorganisms [9, 13]. However, RVS extracts detoxified by solvents and irradiation are not suitable for use in food.

We have been studying the biological activities of RVS along with many other researchers, and to date, several bioactivities have been reported. However, studies on detoxified RVS extracts are still inadequate.

To the best of our knowledge, there are no studies comparing RVS extracts prepared by different detoxification methods. Thus, the present study was conducted to compare the bioactive constituents and biological activities of three RVS extracts prepared by different detoxification methods. The investigated extracts (DRVE, FRVE, and FFRVE) are commercially available as allergen-free functional food in Korea.

The antioxidant activities of the three extracts were assessed via DPPH scavenging assay. Among the three extracts, DRVE was found to possess a superior antioxidant activity. Regarding antimicrobial activity, FRVE was the most effective, and inhibited the growth of both *P. acnes* and *T. rubrum*. The three extracts successfully suppressed hepatic lipogenesis in an in vitro model of non-alcoholic fatty liver. However, DRVE and FRVE were more effective than FFRVE at the same concentration. These results suggest that the different detoxification methods may induce alterations in the major components of RVS, leading to differences in their activities. Therefore, we analyzed the polyphenolic constituents of RVS in the three extracts.

Polyphenols are bioactive compounds present at high concentrations in various plants [18]. Many studies have reported that phenolics such as a fustin, fisetin, gallic acid, and quercetin are highly abundant compounds in *R. verniciflua* [3, 19, 20].

According to a previous report, RVS extracts detoxified via heating methods show high gallic acid contents [17]. This is consistent with our finding that DRVE, an allergen-free RVS extract detoxified by heating to a high temperature, possesses the highest gallic acid content. Moreover, another study reported that an allergen-free RVS extract detoxified by heating (by roasting in an iron pot at 240 °C for 50 min and extracting with water) contains higher amounts of fustin (130 mg/g) than fisetin (20 mg/g) [21]. Similar to these data, our results demonstrated that DRVE contained three times more fustin than fisetin. However, FFRVE, the RVS extract detoxified by fermentation with *F. fraxinea* mushroom, was found to contain more fisetin than fustin*.* This result is in agreement with that reported in a previous study [7]. Finally, FRVE, the RVS extract detoxified by fermentation with the yeast *S. carlsbergensis*, contained higher levels of fustin than fisetin or gallic acid, unlike DRVE or FFRVE. As shown in Tables 2 and 3, DRVE was found to contain the highest amount of gallic acid among the three extracts, and gallic acid was found to be the second most effective antioxidant after fisetin. Therefore, gallic acid was considered to be a marker compound reflecting the antioxidant effect of DRVE, while fustin was considered to be a marker compound reflecting the bioactivity of FRVE. However, the contents of all marker compounds, including fisetin, were low. Therefore, FFRVE seems to have a low biological activity when compared to DRVE or FRVE.

## Conclusions

The present study reports a comparison between the components and biological activities of three different kinds of commercially available allergen-free RVS extracts in Korea. Our findings suggest that the components may vary according to the detoxification method used. Accordingly, by altering the detoxification method, it is possible to maximize the concentrations of components that exert specific effects for application in the health food or cosmetic product industry.

## Abbreviations

AMPK: AMP-activated protein kinase
D.W: Distilled Water
DMSO: Dimethyl sulfoxide
DPPH: 1,1-diphenyl-2-picrylyhdrazyl
DRVE: Dried *Rhus verniciflua STOKES* extract
EC_50_: Half maximal effective concentration
FBS: Fetal Bovine Serum
FFRVE: Fermented with Fomitella *fraxinea Rhus verniciflua* STOKES extract
FRVE: Fermented *Rhus verniciflua* STOKES extract
HRP: Horseradish peroxidase
MTT: 3-(4–5-dimethylthiazol-2-yl)-2,5-diphenyle tetrazolium bromide
O.D: Optical density
OA: Oleic acid
PBS: Phosphate-buffered saline; milk
PPARPα: Peroxisome proliferator-activated receptor alpha
RVS: *Rhus verniciflua* STOKES
SD: Standard deviation
SREBP-1: Sterol regulatory element-binding protein 1
TG: Triglyceride
